# Motivation to Have COVID-19 Vaccination Explained Using an Extended Protection Motivation Theory among University Students in China: The Role of Information Sources

**DOI:** 10.3390/vaccines9040380

**Published:** 2021-04-13

**Authors:** Peng-Wei Wang, Daniel Kwasi Ahorsu, Chung-Ying Lin, I-Hua Chen, Cheng-Fang Yen, Yi-Jie Kuo, Mark D. Griffiths, Amir H. Pakpour

**Affiliations:** 1 Department of Psychiatry, School of Medicine College of Medicine, Kaohsiung Medical University, Kaohsiung 807, Taiwan; 990107@gap.kmu.edu.tw; 2 Department of Psychiatry, Kaohsiung Medical University Hospital, Kaohsiung 807, Taiwan; 3 Department of Rehabilitation Sciences, Faculty of Health & Social Sciences, The Hong Kong Polytechnic University, Hung Hom, Hong Kong, China; daniel.ahorsu@connect.polyu.hk; 4 Institute of Allied Health Sciences, College of Medicine, National Cheng Kung University, Tainan 701, Taiwan; cylin36933@gs.ncku.edu.tw; 5 Department of Occupational Therapy, College of Medicine, National Cheng Kung University, Tainan 701, Taiwan; 6 Department of Public Health, National Cheng Kung University Hospital, College of Medicine, National Cheng Kung University, Tainan 701, Taiwan; 7 School of Education Science, Minnan Normal University, Zhangzhou 363000, China; 8 Key Laboratory of Applied Cognition & Personality, Fujian Province University, Zhangzhou 363000, China; 9 International College, Krirk University, Bangkok 10220, Thailand; 10 Department of Orthopedic Surgery, Wan Fang Hospital, Taipei Medical University, Taipei 110, Taiwan; benkuo5@tmu.edu.tw; 11 Department of Orthopedic Surgery, School of Medicine, College of Medicine, Taipei Medical University, Taipei 110, Taiwan; 12 International Gaming Research Unit, Psychology Department, Nottingham Trent University, Nottingham NG1 4FQ, UK; mark.griffiths@ntu.ac.uk; 13 Department of Nursing, School of Health and Welfare, Jönköping University, 55318 Jönköping, Sweden; apakpour@qums.ac.ir

**Keywords:** COVID-19, vaccination, protection motivation theory, motivation, information sources

## Abstract

Background: The aims of the present study were to examine the prediction of the threat and coping appraisal utilizing an extended protection motivation theory (PMT) for the motivation to have COVID-19 vaccination and the influence of various information sources on coping appraisal among university students in China. Methods: The sample comprised 3145 students from 43 universities in China who completed an online survey including PMT constructs as well as constructs added to PMT. The PMT constructs comprised motivation to have COVID-19 vaccination, threat appraisal, and coping appraisal. The extended PMT constructs comprised knowledge about mechanisms and information sources of COVID-19 vaccination. Results: Perceived severity of COVID-19 was positively associated with motivation to have COVID-19 vaccination. Receiving information concerning COVID-19 vaccination from medical personnel was associated with greater self-efficacy, response efficacy, and knowledge, whereas receiving information concerning COVID-19 vaccination from coworkers/colleagues was associated with less response efficacy and knowledge. Receiving online information concerning COVID-19 vaccination was associated with greater response cost of vaccination efficacy and less knowledge. Conclusions: This study supported the prediction of perceived severity in the PMT for motivation to have COVID-19 vaccination among university students in China. Vaccination information sources have different effects on students’ coping appraisal of COVID-19 vaccination.

## 1. Introduction

The coronavirus disease 2019 (COVID-19) pandemic has continued to impact multiple domains of human lives since its emergence at the end of 2019 [1,2,3,4,5,6]. At the time of writing (April 2021), over 134 million confirmed cases and over 2.9 million deaths had been reported [7]. Vaccination has become the most anticipated intervention in minimizing the spread of COVID-19. Although the development of COVID-19 vaccines worldwide has been accelerated [8], hesitance by some individuals in receiving COVID-19 vaccination is prevalent worldwide [9,10,11,12,13,14,15,16,17]. It is of utmost importance to investigate factors that predict the motivation to have COVID-19 vaccination, especially psychological factors that can be influenced through interventions.

In order to use psychological factors effectively to enhance an individual’s acceptance to vaccination, the use of applied theory is important. Webb and Sheeran synthesized the empirical evidence concerning health behavior changes and found that the use of a theory can assist the healthcare providers in designing an effective intervention for behavior change [18]. Therefore, using a theory to examine the motivation to have COVID-19 vaccination is important in the current global situation. Several theories, such as protection motivation theory (PMT) and theory of planned behavior (TPB) have been found useful in explaining influenza vaccination intention and behaviors [19]. Moreover, the efficacy of PMT on influenza vaccination intention is promising given that Ling et al. reported PMT explained 62% of the variance in vaccination uptake intention [20].

Research has applied PMT [21,22] to examine individuals’ cognitive factors contributing to motivation to have vaccination for respiratory infectious diseases (RIDs) including influenza, measles, mumps, rubella, and pertussis [23,24,25]. According to PMT, threat appraisal and coping appraisal are two major cognitive processes that determine individuals’ motivation to adopt protective behaviors to reduce the risk of contracting RIDs [23]. Threat appraisal depends on the perceived severity of the health threat caused by RIDs and on the perceived vulnerability to RIDs. Coping appraisal depends on the perceived response efficacy (i.e., evaluation of whether self-protective behaviors are effective in alleviating the threat of RIDs) and on perceived self-efficacy (i.e., evaluation of whether the individual will be able to carry out self-protective behaviors) [23,24]. Research has also incorporated the concept of response costs within the concept of coping appraisal [26]. Response costs indicate costs such as money, time, and effort associated with engaging in self-protective behaviors to reduce the threats from RIDs [23].

A systematic review confirmed that perceived high severity, high vulnerability, high response efficacy, high self-efficacy, and low response costs contributed to high motivation to have vaccination for influenza during the 2009 pandemic [23]. Research applying PMT has also found that perceived severity and self-efficacy significantly predicted greater levels of staying at home [27] and intention to self-isolate during the COVID-19 pandemic [28].

The application of PMT in explaining the motivation to have COVID-19 vaccination warrants further studies for several reasons. First, although a recent Swiss study applying PMT demonstrated that individuals show a unified way of determining their motivation to have six different vaccinations [25], it is uncertain whether all constructs of threat and coping appraisals in PMT similarly contributed to individuals’ motivation to have COVID-19 vaccination. COVID-19 vaccines have been developed very quickly to help overcome the COVID-19 pandemic. Individuals may experience more fear of COVID-19 than other RIDs but know less about the benefits and costs of receiving COVID-19 vaccination than other vaccinations. These contextual differences may result in various relationships of threat and coping appraisals in PMT with the motivation to have COVID-19 vaccination compared with other vaccinations. Second, in addition to the components of coping appraisals in PMT, knowledge concerning the main vaccine designs and concepts of protection may be associated with reduced vaccine hesitancy [29]. Whether greater knowledge about the mechanism of COVID-19 vaccination predicts greater motivation to be vaccinated warrants further study.

Third, research has shown that the sources from which individuals obtain information on vaccination play a crucial role in their attitudes and behaviors in relation to vaccination [30,31,32]. A meta-analysis showed that lack of adequate information from health practitioners predicts a lower vaccination acceptance [33]. In order to understand individuals’ behavioral intentions using a more holistic approach, PMT has been extended to include information sources into the analysis [26]. Information sources cover informal and formal sources as well as interpersonal contacts and media coverage [24]. Research examining measles-mumps-rubella (MMR) vaccination demonstrated that parents take formal and informal information concerning the vaccination from both online and offline sources into account. This may have different effects on parents’ coping appraisal and their subsequent behavioral intention to adhere to official MMR vaccination recommendations [30,31]. Individuals may rely on information from the media and/or public discussion to learn about the benefits of COVID-19 vaccination. However, there is a considerable amount of online COVID-19 information that lacks scientific rigor [34]. Whether various sources of information concerning COVID-19 vaccination predict different levels of coping appraisals (which subsequently influence the motivation to have COVID-19 vaccination) warrants further study.

University students experience a variety of challenges during the COVID-19 pandemic; they may experience interruption of academic learning and routine lives on campus, lose on-campus jobs, disconnect from friends and partners, and struggle with the isolation in lockdown or quarantine [35]. Research has demonstrated that mental health problems are prevalent among university students [36,37,38]. However, university students may have low intention to vaccinate because they are not generally clinically vulnerable to COVID-19 [9]. Therefore, understanding psychological factors of motivation to have COVID-19 vaccination among university students may be useful in planning adequate educational strategies for them.

**Figure 1:**
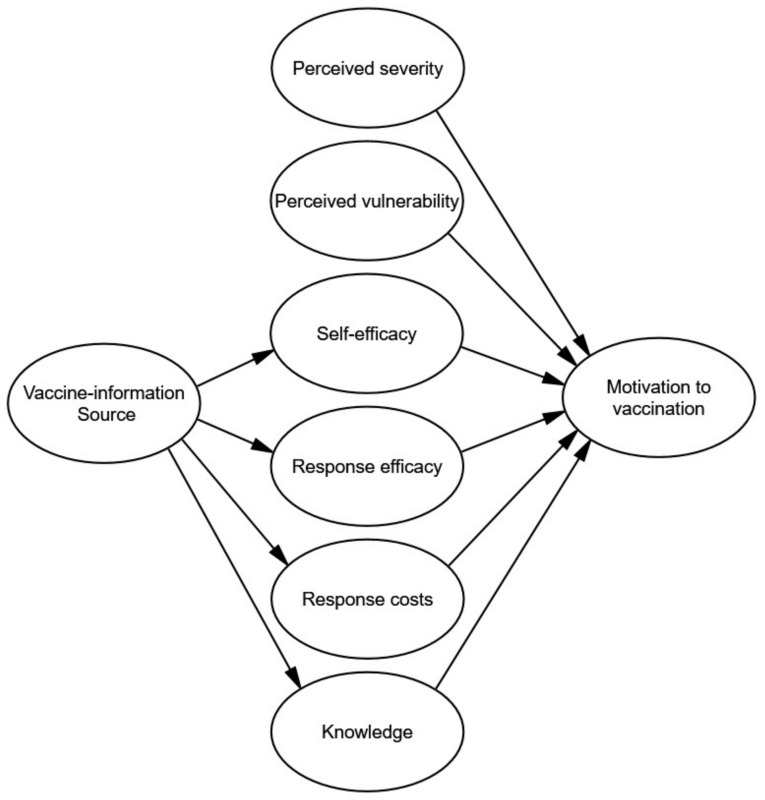
Hypothetic frame.

Based on the PMT, all factors and derived hypotheses of the extended PMT model are summarized in Figure 1. The present study aimed to examine three research questions. First, are threat appraisal (i.e., perceived severity of COVID-19 and perceived vulnerability to COVID-19), coping appraisal (i.e., self-efficacy to have COVID-19 vaccination, response efficacy, and costs of COVID-19 vaccination), and knowledge about the mechanisms of COVID-19 vaccination significantly associated with the motivation to have COVID-19 vaccination among university students in China? Second, do various information sources concerning COVID-19 vaccination have different influences on the self-efficacy, response efficacy, response costs and knowledge of COVID-19 vaccination? Furthermore, do self-efficacy, response efficacy, response costs, and knowledge mediate the association between information sources and motivation to have COVID-19 vaccination? Accordingly, the specific hypotheses are listed below.

**Hypothesis 1(H1)**. *The motivation to have COVID-19 vaccination will have significant positive relationships with perceived severity of COVID-19, perceived susceptibility to COVID-19, perceived efficacy of the vaccine (response efficacy), confidence in an individual’s ability to obtain a vaccination (self-efficacy), and knowledge about the mechanisms of vaccination, as well as a significant negative relationship with perceived response costs of COVID-19 vaccination.*

**Hypothesis 2a(H2a)**. *Various information sources concerning COVID-19 vaccination will have different associations with various components of coping appraisal and knowledge.*

**Hypothesis 2b(H2b)**. *Self-efficacy, response efficacy, response costs and knowledge will mediate the association between information sources and motivation to have COVID-19 vaccination.*

## 2. Materials and Methods

### 2.1. Participants and Procedure

The inclusion criteria for participant eligibility were that individuals had to be (i) studying in a university in mainland China during the survey period, and (ii) young adults (i.e., aged between 18 and 35 years). The study utilized an online survey. The research team engaged the help of college counselors in different universities to help disseminate the online survey to eligible participants via their respective online social communities. The survey period was between 5 and 16 January 2021, and the study protocol was approved by the Institutional Review Board of the Jianxi Psychological Consultant Association (IRB ref: JXSXL-2020-DE22) before the research team approached the college counselors. A total of 3145 students from 43 universities voluntarily participated and all of them provided online informed consent. All the survey items were completed with no missing data because the online survey could only be submitted when all the items were answered. Moreover, the sample size of 3145 achieved sufficient power from the a priori sample size calculation with the following considerations: type I error at 0.05; power at 0.95; degrees of freedom at 15; null RMSEA at 0; and alternative RMSEA at 0.05. From the aforementioned considerations, 750 participants were deemed to be sufficient.

### 2.2. Measures

The measures used in the present study are listed in Table 1.

#### 2.2.1. Protection Motivation Theory (PMT) Measures

The PMT measure included threat appraisal (two items for perceived severity of COVID-19 and four items for perceived vulnerability to COVID-19), self-efficacy in having COVID-19 vaccination (one item), response efficacy of COVID-19 vaccination (six items), response cost of COVID-19 vaccination (three items) and knowledge about the mechanism of COVID-19 vaccination (three items). A higher score on each item indicates a higher level of threat appraisal, self-efficacy, response efficacy, response cost, and knowledge.

#### 2.2.2. Sources of Information Concerning COVID-19 Vaccination

There were seven sources of COVID-19 vaccination information identified in the present study. These were via the internet, traditional media (e.g., television news or newspaper), friends, family, coworkers/classmates, on-site training events concerning COVID-19 (e.g., COVID-19 workshops held by the university), and medical personnel. Each source was assessed using one item rated on a three-point Likert scale. A higher score on each item indicates receiving COVID-19 vaccination information more from that source.

#### 2.2.3. Motivation to Have COVID-19 Vaccination

Motivation to have COVID-19 vaccination was assessed using two items. A higher score on each item indicates a higher level of motivation to have COVID-19 vaccination.

#### 2.2.4. Background Information Questions

As well as the aforementioned PMT variables, the online survey asked several demographic questions, including the participants’ age, program of study (health-related or non-health-related), and level of education (undergraduate or postgraduate).

### 2.3. Data Analysis

Means, medians, and frequencies were calculated to understand the descriptive statistics of participants’ characteristics and their scores on the studied variables. One-sample *t*-tests and χ^2^ tests were used to compare the characteristics of the present sample with those of the Chinese university students. Pearson correlation coefficients were then used to examine the bivariate associations between the studied variables that were tested in the proposed model. Because of multiple comparisons made in the Pearson correlations, the significance level for the Pearson correlations was adjusted using Bonferroni method with *p* < 0.0005 indicating significant correlation. Structural equation modelling (SEM) using diagonally weighted least squares estimator was utilized to examine whether the collected data fit with the proposed model. More specifically, different sources of COVID-19 vaccination information were proposed to be the explanatory factors of PMT components (including self-efficacy to have COVID-19 vaccination, response efficacy, and cost of COVID-19 vaccination), except for the threat appraisal (i.e., perceived severity of COVID-19 and perceived vulnerability to COVID-19), and knowledge about the mechanism of COVID-19 vaccination. All PMT components and knowledge were proposed to be the explanatory factors of the motivation to have COVID-19 vaccination. Therefore, PMT components (except for the threat appraisal) and knowledge were treated as mediators in the proposed model, and the mediation effects were tested using the Sobel test to examine whether such effects were significant. To evaluate the model fit, the fit indices included a nonsignificant χ^2^ test (where significance level was set at *p* < 0.05), a comparative fit index (CFI) > 0.95, a Tucker–Lewis index (TLI) > 0.95, a root mean square error of approximation (RMSEA) < 0.06, and a standardized root mean square residual (SRMR) < 0.08 [39]. For mediated effects, a *t*-value higher than 1.96 indicates significance. Moreover, the proposed model was tested for the entire participants; then, stratified according to the study major (i.e., major in a health-related program and major in a non-health-related program). The SEM was conducted using LISREL 8.7.

## 3. Results

Characteristics of the participants are shown in Table 2. The present sample was relatively young (mean age = 20.80 years [SD = 2.09]) given that most of them were undergraduates (*n* = 3062; 96.2%). Moreover, the majority of the participants were studying in a major subject not related to the health field (*n* = 2904; 92.3%) and slightly over half of the participants were females (*n* = 1578; 50.2%). As compared with the university students in China, the present sample was slightly older (20.80 years vs. 20.00 years; *t* = 19.12; *p* < 0.001), but comparable in gender distribution (χ^2^ = 0.08; *p* = 0.77), educational level (χ^2^ = 1.42; *p* = 0.23), and study major (χ^2^ = 0.31; *p* = 0.58). Table 2 additionally presents the participants’ average scores on all the studied variables, including COVID-19 vaccination information sources, perceived severity, perceived vulnerability, self-efficacy, response efficacy, response cost, knowledge, and motivation to vaccination.

The zero-order bivariate correlations between the studied variables are presented in Table 3. Most of the correlations were significant, except for (i) correlations between perceived vulnerability and three COVID-19 information sources (i.e., from traditional media, on-site training, and medical personnel); (ii) the correlation between perceived vulnerability and motivation to have COVID-19 vaccination; (iii) correlations between perceived severity and two types of efficacy (i.e., self-efficacy and response efficacy); (iv) correlations between response cost and the following variables: self-efficacy, response efficacy, knowledge, and motivation to have COVID-19 vaccination; and (v) correlations between knowledge and the following variables: COVID-19 information sources from internet and coworkers/classmates.

The tested model had satisfactory data-model fit (CFI = 0.999; TLI = 0.993; RMSEA = 0.031; SRMR = 0.042), except for the significant χ^2^ test (χ^2^ = 60.27; df = 15; *p* < 0.001). With the supported fit indices, path coefficients of the tested model were examined (Table 4). Regarding the part of the original PMT, only perceived severity was significantly associated with motivation to have COVID-19 vaccination (standardized coefficient [β] = 0.190; *p* < 0.001). Self-efficacy, response efficacy, response cost, and knowledge were not significantly associated with motivation to have COVID-19 vaccination. The results only partially supported H1.

Receiving information concerning COVID-19 vaccination from medical personnel to self-efficacy to have COVID-19 vaccination (β = 0.115; *p* = 0.01), information from coworkers/classmates to response efficacy of vaccination (β = −0.088; *p* = 0.042), information from medical personnel to response efficacy (β = 0.145; *p* < 0.001), information from the internet to response cost of vaccination (β = 0.151; *p* < 0.001), information from on-site training to response cost (β = 0.170; *p* < 0.001), information from the internet to knowledge (β = −0.226; *p* < 0.001), information from traditional media to knowledge (β = 0.098; *p* < 0.001), information from coworkers/classmates to knowledge (β = −0.94; *p* = 0.027), information from academic training to knowledge (β = 0.121; *p* < 0.001), and information from medical personnel to knowledge (β = 0.102; *p* < 0.001). These results supported H2a.

Given that self-efficacy, response efficacy, response costs and knowledge were not significantly associated with motivation to have COVID-19 vaccination, they did not mediate the association between information sources and motivation to have COVID-19 vaccination. The results did not support H2b.

SEM was further conducted to investigate the proposed model among university students in health-related and non-health-related programs separately. Similar findings were not replicated in those who were majoring in a health-related program (Table S1). All the coefficients and mediation effects were not significant in the subsample majoring in a health-related program. However, findings from those who were majoring in a non-health-related program were similar to those form the entire sample (Table S2).

## 4. Discussion

The present study found that perceived severity of COVID-19 was positively associated with motivation to have a COVID-19 vaccination. Several information sources of COVID-19 vaccination were significantly associated with the PMT components and knowledge concerning the mechanism of COVID-19 vaccination, although the directions of these associations varied.

### 4.1. PMT Components and Motivation to Have a COVID-19 Vaccination

The present study found that perceived severity of COVID-19 was positively associated with motivation to have COVID-19 vaccination. China had the first COVID-19 outbreak in the world. Although the spread of the virus was well controlled after implementing a series of actions including lockdown and quarantine of suspected cases, individuals in China experienced the earliest threats concerning COVID-19. The prevalence rates of symptoms of anxiety, depression, post-traumatic stress disorder, and psychological distress were relatively high in the general population during the initial stages of the COVID-19 pandemic in China [40]. These negative mood experiences may contribute individuals’ motivation to have COVID-19 vaccination. The result of the present study indicated that increasing individuals’ perceived severity of COVID-19 may be effective way to enhance motivation of having a COVID-19 vaccination. However, exposure to images of illness and news of fear may intensify anxiety and result in maladaptive behaviors, such as dismissal or denial, because individuals lack confidence to respond to the threat directly [41]. Governments and health professionals should actively promote awareness among the public regarding the threat of COVID-19 without evoking excessive worry. Research has suggested that the programs of promoting COVID-19 vaccination can apply narrative communication as a tool (e.g., inviting the patients to convey the severity of contracting COVID-19 [27].

Conversely, the components of coping appraisal in PMT (i.e., self-efficacy to have COVID-19 vaccination, response efficacy, and costs of COVID-19 vaccination) and knowledge concerning the mechanism of COVID-19 vaccination did not significantly predict the motivation to have COVID-19 vaccination. Unlike other vaccines that were developed over a long period (often many years), the COVID-19 vaccines were developed very quickly. Although governments around the world expect vaccination to minimize the spread of COVID-19, individuals may feel uneasy about COVID-19 vaccination and may be unsure about vaccination efficacy in mitigating the harm posed by COVID-19 and personal ability to have COVID-19 vaccination. Individuals may also have little understanding of adverse effects of COVID-19 vaccination. Given that individuals may activate a threat appraisal first and a coping appraisal later when they are confronted with a threat [22], individuals may just be at the initial stage of appraising the costs and benefits of having COVID-19 vaccination. Further study is needed to evaluate the prediction of coping appraisal in the motivation of COVID-19 vaccination uptake.

### 4.2. Role of Information Sources of COVID-19 Vaccination

Previous studies have shown that receiving vaccination information from formal and informal sources may have different effects on individuals’ coping appraisal of vaccination [30,31]. The present study had similar results. Receiving information concerning COVID-19 vaccination from medical personnel was associated with greater self-efficacy to obtain vaccination, response efficacy of vaccination, and knowledge about the mechanism of vaccination, whereas receiving information from coworkers/colleagues was associated with less response efficacy and knowledge. The findings further supported those of a recent meta-analysis which highlighted the importance of the type of information and information source for individuals’ immunization outcomes [33].

It is worth noting that receiving online information concerning COVID-19 vaccination was associated with greater response cost of vaccination efficacy and less knowledge. The internet has been a major source of COVID-19 information, especially during the period of lockdown. However, a lot of online information concerning COVID-19 lacks scientific rigor [34]. Online information sources are important for individuals in establishing their coping appraisal toward vaccination [30,31]. Therefore, to avoid bias in news spread via the internet, it is crucial for governments to invite medical personnel to introduce the benefits and possible costs of COVID-19 vaccination by both online and traditional media.

### 4.3. Limitations

The present study had a few limitations that should be noted. First, the participants in the study were recruited via convenience sampling, which restricts the generalizability of the present findings. The use of this sampling method also meant the number of students approached was unknown (i.e., the number of participants who received the survey link) and the response rate was unable to be estimated. Second, given that information concerning COVID-19 vaccination is released on a daily basis, university students’ threat and coping appraisals, knowledge, and motivation to have COVID-19 vaccination may change over time. The cross-sectional design of the study also limited the possibility to capture such changes. Third, although the participants’ knowledge about the mechanism of COVID-19 vaccination was evaluated, participants’ accuracy of knowledge could not be confirmed. Additionally, the survey did not assess participants’ experiences of previous exposure to COVID-19 and was unable to determine the influence of previous exposure to COVID-19 on their motivation to get vaccinated. In addition to information sources, factors such as physical and mental health that may influence individuals’ threat and coping appraisals and motivation to have COVID -19 vaccination also warrant further study. Finally, the most important limitation of the present study concerns the restriction of internet use in mainland China. The authorities and the government in mainland China prohibit the use of some websites (e.g., Google); therefore, participants in the present study might not have full freedom to obtain all the COVID-19-related information they require. In this regard, the hypotheses relating to information sources concerning COVID-19 vaccination may not have been correctly answered by the present study’s participants. Therefore, future studies are warranted among other samples (e.g., individuals in countries such as the US and the UK) to corroborate the present study’s findings.

## 5. Conclusions

The present study found that perceived severity of COVID-19 significantly predicted university students’ motivation to have COVID-19 vaccination in China. Receiving information of COVID-19 vaccination from medical personnel, the internet, and coworkers/colleagues had different effects on university students’ coping appraisal and knowledge of vaccination. Based on the results, it is recommended that government health departments invite medical personnel to introduce COVID-19 threats in a balanced way to promote individuals’ awareness as well as the benefits and costs of receiving vaccination to enhance individuals’ appraisal of COVID-19 vaccination. It is also recommended that further studies based on PMT are needed to examine other factors related to motivation and behaviors that would increase the uptake of COVID-19 vaccination.

## Figures and Tables

**Table 1:** Measures used in the present study.

Measures	Items	Response Scale
Threat appraisal	Item 1: Please rate the current level of your worry towards COVID-19:	1 (very mild) to 10 (very severe)
	Item 2: How serious is COVID-19 relative to SARS?	1 (much less serious) to 5 (much more serious)
	Item 3: How likely do you think it is that you will contract COVID-19 over the next 1 month?	1 (never) to 7 (certain)
	Item 4: If you were to develop flu-like symptoms tomorrow, would you be:	1 (not at all worried) to 7 (extremely worried)
	Item 5: In the past one week, have you ever worried about catching COVID-19?	1 (no, never think about it) to 5 (worried about it all the time)
	Item 6: What do you think are your chances of getting COVID-19 over the next 1 month compared to others outside your family?	1 (not at all) to 7 (certain)
Self-efficacy in having COVID-19 vaccination	I can choose whether to get a COVID-19 jab or not.	1 (strongly disagree) to 7 (strongly agree)
Response efficacy of COVID-19 vaccination was assessed using six items	Item 1: Vaccination is a very effective way to protect me against COVID-19.	1 (strongly disagree) to 7 (strongly agree)
	Item 2: It is important that I get the COVID-19 jab.	
	Item 3: Vaccination greatly reduces my risk of catching COVID-19.	
	Item 4: The COVID-19 jab plays an important role in protecting my life and that of others.	
	Item 5: The contribution of the COVID-19 jab to my health and well-being is very important.	
	Item 6: Getting the COVID-19 jab has a positive influence on my health.	
Response cost of COVID-19 vaccination	(1) Safety and possible side effects of vaccine; (2) cost of vaccine of vaccine; and (3) time spent on vaccination will influence my willingness to get COVID-19 vaccinated.	1 (strongly disagree) to 4 (strongly agree)
Knowledge about the mechanism of COVID-19 vaccination	Item 1: I understand how the COVID-19 jab helps my body fight the COVID-19 virus.	1 (strongly disagree) to 7 (strongly agree) *: Reverse scoring
	Item 2: I know very well how vaccination protects me from COVID-19.	
	Item 3: How the COVID-19 jab works to protect my health is a mystery to me *.	
Sources of information concerning COVID-19 vaccination	Do you obtain COVID-19 vaccination information from (1) internet media (e.g., Facebook, Twitter, blogs, and internet news); (2) friends; (3) traditional media (e.g., newspapers, television, and radio broadcasting); (4) academic courses (e.g., online or in-person formal courses lectured by experts); (5) medical staff in health care institutions; (6) coworkers, and (7) family members?	0 (never) to 2 (always)
Motivation to have COVID-19 vaccination	Item 1: When a COVID-19 vaccine becomes available, will you get vaccinated?	1 (definitely not willing) to 4 (definitely not willing)
	Item 2: Please rate the current level of your willing to receive a COVID-19 vaccine:	1 (very low) to 10 (very high)

**Table 2:** Characteristics of the participants (*N* = 3145).

Characteristics	Mean (SD)	Median (Possible Range)	*N* (%)	Compared with China University Students Χ^2^ or *T* (*p*-Value)
Gender (female)			1578 (50.2)	0.08 (0.77)
Age (years)	20.80 (2.09)	20.00 (18–40)		19.12 (<0.001)
Education level (undergraduate)			3026 (96.2)	1.42 (0.23)
Professional (health-related)			241 (7.7)	0.31 (0.58)
COVID-19 information source				
From the internet	3.54 (0.76)	4 00 (1–4)		
From traditional media	3.03 (0.98)	3 00 (1–4)		
From friends	3.02 (0.88)	3.00 (1–4)		
From family	3.04 (0.89)	3.00 (1–4)		
From coworkers/classmates	3.03 (0.86)	3.00 (1–4)		
From on-site training	2.68 (0.98)	3.00 (1–4)		
From medical personnel	2.57 (1.03)	3.00 (1–4)		
Perceived severity of COVID-19	4.54 (1.65)	4.50 (1–7.5)		
Perceived vulnerability to COVID-19	2.61 (0.84)	2.50 (1–6)		
Self-efficacy to have COVID-19 vaccination	5.78 (1.24)	6.00 (1–7)		
Response efficacy of COVID-19 vaccination	5.82 (0.94)	6.00 (1–7)		
Response cost of COVID-19 vaccination	3.14 (0.68)	3.00 (1–4)		
Knowledge about the mechanism of COVID-19 vaccination	5.34 (0.98)	5.33 (1–7)		
Motivation to vaccination	5.31 (1.44)	5.50 (1–7)		

SD: Standard deviation.

**Table 3:** Pearson correlation in the associations between studied variables.

Studied variables	r													
	1.	2.	3.	4.	5.	6.	7.	8.	9.	10.	11.	12.	13.	14
1. Internet ^a^	1													
2. Traditional media ^a^	**0.492**	1												
3. Friends ^a^	**0.510**	**0.514**	1											
4. Family ^a^	**0.494**	**0.511**	**0.808**	1										
5. Coworkers/classmates ^a^	**0.504**	**0.512**	**0.776**	**0.704**	1									
6. On-site training ^a^	**0.312**	**0.533**	**0.582**	**0.551**	**0.651**	1								
7. Medical personnel ^a^	**0.251**	**0.513**	**0.497**	**0.487**	**0.539**	**0.737**	1							
8. Perceived severity	**0.156**	**0.105**	**0.131**	**0.122**	**0.138**	**0.075**	**0.068**	1						
9. Perceived vulnerability	0.042 ^N^	0.023 ^N^	0.053 ^N^	0.052 ^N^	0.062 ^N^	0.032 ^N^	0.010 ^N^	**0.520**	1					
10. Self-efficacy	0.059 ^N^	**0.092**	**0.087**	**0.074**	**0.073**	**0.090**	**0.109**	−0.039 ^N^	**−0.117**	1				
11. Response efficacy	**0.081**	**0.125**	**0.100**	**0.096**	**0.088**	**0.125**	**0.151**	0.001 ^N^	**−0.130**	**0.558**	1			
12. Response cost	**0.178**	**0.127**	**0.165**	**0.140**	**0.177**	**0.166**	**0.116**	**0.103**	**0.064**	0.014 ^N^	0.007 ^N^	1		
13. Knowledge	−0.058 ^N^	**0.088**	**0.065**	**0.066**	0.051 ^N^	**0.156**	**0.173**	−0.070	**−0.166**	**0.488**	**0.685**	0.057 ^N^	1	
14. Motivation ^b^	**0.219**	**0.187**	**0.157**	**0.167**	**0.163**	**0.145**	**0.138**	**0.178**	0.036 ^N^	**0.280**	**0.487**	0.001 ^N^	**0.229**	1

^a^ COVID-19 information sources; ^b^ Motivation to COVID-19 vaccination; ^N^ Nonsignificant; values in bold are significant at the *p* < 0.0005 level.

**Table 4:** Results of the structural equation modeling investigating the proposed model.

IV	Mediator	DV	Coeff. (SE)	Stand. Coeff.	*T*-Value	*p*-Value
Perceived severity		Motivation	**0.190 (0.032)**	**0.190**	**5.978**	**<0.001**
Perceived vulnerability		Motivation	0.000 (0.024)	0.000	0.007	0.994
Internet ^a^	Self-efficacy		0.000 (0.049)	0.000	0.001	0.999
Traditional media ^a^	Self-efficacy		0.046 (0.041)	0.046	1.137	0.255
Friends ^a^	Self-efficacy		0.075 (0.062)	0.075	1.209	0.226
Family ^a^	Self-efficacy		−0.016 (0.054)	−0.016	−0.298	0.765
Coworkers/classmates ^a^	Self-efficacy		−0.049 (0.053)	−0.049	−0.932	0.351
On-site training ^a^	Self-efficacy		0.000 (0.049)	0.000	0.007	0.994
Medical personnel ^a^	Self-efficacy		**0.115 (0.045)**	**0.115**	**2.526**	**0.010**
Internet ^a^	Response efficacy		0.064 (0.036)	0.064	1.775	0.076
Traditional media ^a^	Response efficacy		0.039 (0.035)	0.039	1.120	0.262
Friends ^a^	Response efficacy		0.033 (0.052)	0.033	0.635	0.525
Family ^a^	Response efficacy		−0.012 (0.045)	−0.012	−0.258	0.796
Coworkers/classmates ^a^	Response efficacy		**−0.088 (0.044)**	**−0.088**	**−2.026**	**0.042**
On-site training ^a^	Response efficacy		0.020 (0.041)	0.020	0.491	0.622
Medical personnel ^a^	Response efficacy		**0.145 (0.037)**	**0.145**	**3.911**	**<0.001**
Internet ^a^	Response cost		**0.151 (0.047)**	**0.151**	**3.214**	**<0.001**
Traditional media ^a^	Response cost		−0.048 (0.039)	−0.048	−1.246	0.212
Friends ^a^	Response cost		0.062 (0.058)	0.062	1.055	0.291
Family ^a^	Response cost		−0.083 (0.050)	−0.083	−1.671	0.094
Coworkers/classmates ^a^	Response cost		0.035 (0.054)	0.035	0.657	0.510
On-site training ^a^	Response cost		**0.170 (0.047)**	**0.170**	**3.622**	**<0.001**
Medical personnel ^a^	Response cost		−0.033 (0.041)	−0.033	−0.798	0.424
Internet ^a^	Knowledge		**−0.226 (0.036)**	**−0.226**	**−6.249**	**<0.001**
Traditional media ^a^	Knowledge		**0.098 (0.032)**	**0.098**	**3.037**	**<0.001**
Friends ^a^	Knowledge		0.076 (0.049)	0.076	1.557	0.119
Family ^a^	Knowledge		0.032 (0.041)	0.032	0.772	0.440
Coworkers/classmates ^a^	Knowledge		**−0.094 (0.043)**	**−0.094**	**−2.199**	**0.027**
On-site training ^a^	Knowledge		**0.121 (0.040)**	**0.121**	**2.989**	**<0.001**
Medical personnel ^a^	Knowledge		**0.102 (0.036)**	**0.102**	**2.802**	**<0.001**
	Self-efficacy	Motivation ^b^	0.121 (0.382)	0.121	0.316	0.752
	Response efficacy	Motivation ^b^	0.542 (0.875)	0.542	0.620	0.535
	Response cost	Motivation ^b^	−0.018 (0.029)	−0.018	−0.633	0.527
	Knowledge	Motivation ^b^	−0.179 (0.359)	−0.179	−0.499	0.618

^a^ COVID-19 information sources; ^b^ Motivation to COVID-19 vaccination; IV = independent variable; DV = dependent variable; Coeff. = coefficient; SE = standard error; Stand. Coeff. = standardized coefficient. Significant values are in bold.

## Data Availability

The data will be available upon reasonable request to the corresponding authors.

## Supplementary Materials

The following are available online at https://www.mdpi.com/article/10.3390/vaccines9040380/s1, Table S1: Results of the structural equation modeling investigating the proposed model (participants with a health-related major), Table S2: Results of the structural equation modeling investigating the proposed model (participants with a non-health-related major).
