# Supplementary material for: Motivation to Have COVID-19 Vaccination Explained Using an Extended Protection Motivation Theory among University Students in China: The Role of Information Sources
Source: Vaccines (Basel). 2021 Apr 13;9(4):380. doi: 10.3390/vaccines9040380 (PMC8070343; doi:10.3390/vaccines9040380)
Supplement: Supplementary file 1 [file vaccines-09-00380-s001.pdf]

Table S1. Results of the structural equation modeling investigating the proposed model (participants with a health-related major).

IV	Mediator	DV	Coeff. (SE)	Stand. Coeff.	t-value	*p*-value
Perceived severity		Motivation	0.204 (0.617)	0.204	0.330	0.741
Perceived vulnerability		Motivation	0.004 (1.284)	0.004	0.003	0.997
Internet[a]	Self-efficacy		0.100 (0.203)	0.100	0.494	0.621
Traditional media[a]	Self-efficacy		−0.125 (0.160)	−0.125	−0.783	0.434
Friends[a]	Self-efficacy		−0.063 (0.189)	−0.063	−0.334	0.738
Family[a]	Self-efficacy		0.027 (0.149)	0.027	0.182	0.856
Coworkers/classmates[a]	Self-efficacy		−0.017 (0.152)	−0.017	−0.114	0.909
On-site training[a]	Self-efficacy		0.091 (0.209)	0.091	0.436	0.663
Medical personnel[a]	Self-efficacy		0.182 (0.191)	0.182	0.956	0.339
Internet[a]	Response efficacy		0.041 (0.224)	0.041	0.183	0.855
Traditional media[a]	Response efficacy		0.084 (0.129)	0.084	0.655	0.513
Friends[a]	Response efficacy		−0.115 (0.189)	−0.115	−0.609	0.543
Family[a]	Response efficacy		0.014 (0.131)	0.014	0.110	0.912
Coworkers/classmates[a]	Response efficacy		0.109 (0.149)	0.109	0.735	0.462
On-site training[a]	Response efficacy		−0.016 (0.136)	−0.016	−0.119	0.905
Medical personnel[a]	Response efficacy		0.114 (0.130)	0.114	0.874	0.382
Internet[a]	Response cost		−0.101 (0.198)	−0.101	−0.510	0.610
Traditional media[a]	Response cost		−0.124 (0.143)	−0.124	−0.863	0.388
Friends[a]	Response cost		0.085 (0.187)	0.085	0.454	0.649
Family[a]	Response cost		0.070 (0.165)	0.070	0.421	0.673
Coworkers/classmates[a]	Response cost		0.267 (0.179)	0.267	1.496	0.134
On-site training[a]	Response cost		0.029 (0.144)	0.029	0.201	0.841
Medical personnel[a]	Response cost		−0.051 (0.134)	−0.051	−0.377	0.706
Internet[a]	Knowledge		−0.221 (0.214)	−0.221	−1.031	0.302
Traditional media[a]	Knowledge		−0.005 (0.132)	−0.005	−0.039	0.968
Friends[a]	Knowledge		0.078 (0.195)	0.078	0.401	0.688
Family[a]	Knowledge		−0.086 (0.131)	−0.086	−0.661	0.508
Coworkers/classmates[a]	Knowledge		0.179 (0.168)	0.179	1.065	0.286
On-site training[a]	Knowledge		0.024 (0.153)	0.024	0.160	0.872
Medical personnel[a]	Knowledge		0.116 (0.130)	0.116	0.897	0.369
	Self-efficacy	Motivation[b]	0.427 (1.366)	0.427	0.313	0.754
	Response efficacy	Motivation[b]	−0.037 (3.383)	−0.037	−0.011	0.991
	Response cost	Motivation[b]	−0.207 (0.126)	−0.207	−1.645	0.100
	Knowledge	Motivation[b]	0.111 (1.588)	0.111	0.070	0.944

[a] COVID-19 information sources; [b] Motivation to COVID-19 vaccination; IV = independent variable; DV = dependent variable; Coeff. = coefficient; SE = standard error; Stand. Coeff. = standardized coefficient.

Table S2. Results of the structural equation modeling investigating the proposed model (participants with a non-health-related major).

IV	Mediator	DV	Coeff. (SE)	Stand. Coeff.	t-value	*p*-value
Perceived severity		Motivation	**0.190 (0.031)**	**0.190**	**6.179**	**<0.001**
Perceived vulnerability		Motivation	−0.001 (0.024)	−0.001	−0.024	0.981
Internet[a]	Self-efficacy		−0.006 (0.051)	−0.006	−0.127	0.898
Traditional media[a]	Self-efficacy		0.059 (0.043)	0.059	1.389	0.164
Friends[a]	Self-efficacy		0.086 (0.066)	0.086	1.308	0.191
Family[a]	Self-efficacy		−0.016 (0.058)	−0.016	−0.272	0.786
Coworkers/classmates[a]	Self-efficacy		−0.055 (0.056)	−0.055	−0.974	0.330
On-site training[a]	Self-efficacy		0.008 (0.050)	0.008	0.168	0.867
Medical personnel[a]	Self-efficacy		**0.110 (0.047)**	**0.110**	**2.332**	**0.019**
Internet[a]	Response efficacy		0.067 (0.037)	0.067	1.815	0.069
Traditional media[a]	Response efficacy		0.034 (0.036)	0.034	0.941	0.346
Friends[a]	Response efficacy		0.046 (0.055)	0.046	0.851	0.394

Family[a]	Response efficacy		−0.015 (0.048)	−0.015	−0.304	0.761
Coworkers/classmates[a]	Response efficacy		**−0.107 (0.046)**	**−0.107**	**−2.305**	**0.021**
On-site training[a]	Response efficacy		0.026 (0.043)	0.026	0.614	0.539
Medical personnel[a]	Response efficacy		**0.147 (0.039)**	**0.147**	**3.767**	**<0.001**
Internet[a]	Response cost		**0.165 (0.048)**	**0.165**	**3.414**	**<0.001**
Traditional media[a]	Response cost		−0.038 (0.040)	−0.038	−0.944	0.345
Friends[a]	Response cost		0.063 (0.062)	0.063	1.022	0.306
Family[a]	Response cost		−0.100 (0.052)	−0.100	−1.934	0.053
Coworkers/classmates[a]	Response cost		0.018 (0.057)	0.018	0.308	0.758
On-site training[a]	Response cost		**0.184 (0.050)**	**0.184**	**3.705**	**<0.001**
Medical personnel[a]	Response cost		−0.031 (0.043)	−0.031	−0.725	0.468
Internet[a]	Knowledge		**−0.228 (0.037)**	**−0.228**	**−6.155**	**<0.001**
Traditional media[a]	Knowledge		**0.107 (0.034)**	**0.107**	**3.193**	**<0.001**
Friends[a]	Knowledge		0.077 (0.052)	0.077	1.498	0.134
Family[a]	Knowledge		0.038 (0.044)	0.038	0.874	0.382
Coworkers/classmates[a]	Knowledge		**−0.118 (0.045)**	**−0.118**	**−2.608**	**0.009**
On-site training[a]	Knowledge		**0.133 (0.043)**	**0.133**	**3.121**	**<0.001**
Medical personnel[a]	Knowledge		**0.097 (0.038)**	**0.097**	**2.563**	**0.010**
	Self-efficacy	Motivation[b]	0.116 (0.357)	0.116	0.325	0.745
	Response efficacy	Motivation[b]	0.544 (0.837)	0.544	0.650	0.515
	Response cost	Motivation[b]	−0.007 (0.029)	−0.007	−0.253	0.800
	Knowledge	Motivation[b]	−0.181 (0.351)	−0.181	−0.516	0.605

[a] COVID-19 information sources; [b] Motivation to COVID-19 vaccination; IV = independent variable; DV = dependent variable; Coeff. = coefficient; SE = standard error; Stand. Coeff. = standardized coefficient. Significant values are in **bold.**
